# The Hidden Anatomy of Low Back Pain: Uncovering the Impact of Mamillo-Accessory Ligament Ossification

**DOI:** 10.3390/jfmk11010100

**Published:** 2026-02-27

**Authors:** Jordan Allan Piper, Koko Faen, Andy Cai, Ali Ghahreman, Samuel Rajadurai, Giuseppe Musumeci, Alessandro Castorina

**Affiliations:** 1 Laboratory of Cellular and Molecular Neuroscience, School of Life Sciences, Faculty of Science, University of Technology Sydney, Sydney, NSW 2007, Australia; jordan.piper@uts.edu.au (J.A.P.); koko.r.faen@student.uts.edu.au (K.F.); andy.cai@uts.edu.au (A.C.); 2 Department of Neurosurgery, St. George Hospital, Sydney, NSW 2217, Australia; draghahreman@gmail.com (A.G.); samuel.rajadurai@hotmail.com (S.R.); 3 Department of Biomedical and Biotechnological Sciences, Section of Anatomy, Histology and Movement Science, School of Medicine, University of Catania, Via S. Sofia n°97, 95123 Catania, Italy; g.musumeci@unict.it; 4 Research Center on Motor Activities (CRAM), University of Catania, Via S. Sofia n°97, 95123 Catania, Italy

**Keywords:** facetogenic pain, paraspinal muscle dysfunction, entrapment neuropathy, functional spinal anatomy, lumbar biomechanics

## Abstract

Low back pain (LBP) remains a leading cause of disability worldwide, imposing substantial socioeconomic burdens. Among its many causes, facetogenic pain accounts for a significant proportion of cases and is generally attributed to irritation of the richly innervated facet joint capsule, mediated by the medial branches of the dorsal rami. This narrative, hypothesis-driven review synthesises the current anatomical, biomechanical, neurophysiological, and clinical literature and advances a conceptual framework proposing a novel anatomical mechanism that may contribute to LBP. We hypothesise that ossification of the mamillo-accessory ligament (MAL) may be a plausible but under-recognised anatomical variant that may influence lumbar biomechanics and neural interfaces. The MAL connects the mammillary and accessory processes of lumbar vertebrae, serving as a stabilising anchor for deep paraspinal muscles and forming a conduit for the medial branch of the dorsal ramus (MBDR). Ossification of the MAL, resulting in a mamillo-accessory foramen, may theoretically impair spinal biomechanics via three principal mechanistic domains: (1) disruption of muscle attachment and segmental stabilisation, (2) potential compression of the MBDR causing denervation and muscle atrophy, and (3) chronic nerve entrapment leading to asymmetrical postural adaptations and persistent pain. Collectively, these pathways may contribute to spinal instability, facet degeneration, and variable response to standard interventional treatments such as radiofrequency ablation. Recognition of MAL ossification may have potential implications for clinical assessment, targeted imaging strategies, and treatment stratification in patients with chronic, non-specific LBP.

## 1. Introduction

Low back pain (LBP) has been identified as a leading contributor to disability worldwide [1]. Its widespread prevalence and considerable impact on quality of life place a substantial burden not only on individuals but also on healthcare systems and the global economy. As such, LBP is typically considered a major driver of healthcare utilisation, lost productivity, and early retirement, with indirect costs often exceeding direct medical expenses [2,3]. Given its complex and multifactorial nature, along with the heterogeneity of its underlying causes, identifying specific anatomical contributors to LBP is critical for enhancing diagnostic precision and guiding targeted therapeutic interventions.

Among the numerous potential causes of LBP, lumbar facet joints are increasingly recognised as a clinically significant pain generator. Their contribution to LBP has been estimated to range from 4.8% to over 50%, with incidence increasing with age and degenerative change [4]. In contrast, ossification of the mamillo-accessory ligament (MAL) has been documented in cadaveric and radiological studies with prevalence estimates ranging from approximately 10 to 72%, depending on vertebral level and morphological type [5]; however, the true prevalence of clinically significant MAL ossification in symptomatic populations remains unknown due to limited imaging protocols and the absence of large-scale epidemiological studies. As such, a deeper understanding of structural factors that influence facet joint function is crucial to refining therapeutic approaches and reducing the long-term burden of disease.

Biomechanically, each lumbar vertebral level functions as a three-joint complex, comprising an anterior intervertebral disc and a paired posterior facet joint, which together distribute mechanical loads and coordinate spinal motion [6]. Spinal facet joints, or zygapophyseal joints, are synovial articulations formed by the superior and inferior articular processes of adjacent vertebrae [7,8]. These joints play a key role in maintaining spinal stability and directing segmental motion, particularly flexion and extension, while restricting excessive axial rotation [6,8,9]. Each facet joint is innervated by the medial branch of the dorsal ramus (MBDR) of its respective spinal nerve, which transmits sensory and nociceptive afferents from the joint capsule as well as its surrounding tissues [10,11]. When these joints deteriorate in a condition known as facet joint degeneration (FJD), they can become a significant source of chronic LBP, owing to its rich sensory innervation [12]. FJD is often associated with other spinal pathologies such as degenerative disc disease, osteoarthritis, spondylosis, and spondylolisthesis [13,14,15]. While this makes its aetiology both complex and multifactorial, FJD can generally be considered to result from cumulative mechanical stress, microtrauma, age-related changes, and anatomical variation [4]. Consequently, identifying structural factors that may influence facet joint mechanics is critical to understanding the development of FJD and, thus, spinal dysfunction—allowing for more targeted therapeutic strategies for the treatment of LBP.

As mentioned, one such structural element is the MAL, a small fibrous band of dense regular connective tissue found predominantly in the lumbar spine [16]. This ligament is often considered a “false” ligament as its course extends between two regions of the same bone—in this case, from the mammillary process of the superior articular process to the accessory process of the transverse process. While the function of this ligament has not been reported extensively in the literature, its proximity to the intrinsic back muscles allows the ligament to serve as an attachment site for the multifidus and longissimus thoracis [16,17]. This structural connectivity is thought to act as an indirect stabiliser for these muscles and therefore contribute to segmental stability of the lumbar spine during movement and load bearing.

In addition to its muscular associations, the MAL also forms a fibro-osseous arch that creates a tunnel through which the medial branch of the dorsal rami passes [16]. This structural configuration places the nerve in a confined anatomical corridor and allows the nerve to maintain its anatomical trajectory along the vertebral arch. Critically, standard clinical imaging such as X-ray and routine MRI often do not visualise the MAL or its ossification reliably; high-resolution CT and targeted MRI neurography are required to assess MAL morphology and identify ossification, foramina morphology, and associated nerve compression [17]. This fibro-osseous arch can become ossified later in life, creating either a mamillo-accessory notch (MAN) or foramen (MAF) [5,17,18,19]. MAL ossification has been categorised into morphological types (Type I: ½ notch; Type II: ¾ notch; Type III: complete foramen), with the degree of ossification potentially correlating with the severity of biomechanical alterations and nerve compression. MAL ossification of all types is most prevalent at the L3–L5 vertebral levels, with incidence increasing with age [18,19].

Ossification of spinal ligaments such as the posterior longitudinal ligament, ligamentum flavum, and anterior longitudinal ligament has been extensively characterised in the literature, with well-documented effects on spinal biomechanics, segmental motion, and the development of degenerative pathology [20,21,22,23]. In contrast, the MAL has received little attention, largely owing to its small size, limited visibility on standard imaging, and lack of emphasis in clinical training. However, since ossification of other spinal ligaments has been established to alter load distribution, reduce flexibility, and produce clinically significant symptoms, there is a strong rationale to infer that MAL ossification may exert comparable biomechanical and pathological effects.

From a clinical perspective, current management of suspected facetogenic pain includes physiotherapy-based stabilisation programmes, pharmacological therapy, diagnostic medial branch blocks, radiofrequency ablation, and, in selected cases, surgical decompression. However, the potential contribution of MAL ossification is not routinely considered within these diagnostic and therapeutic pathways [24,25].

Given the MAL’s anatomical relationships with stabilising musculature, neural structures, and facet joints, its ossification has the potential to influence spinal biomechanics, paraspinal muscle function, and facet joint mechanics. Although the occurrence and frequency of MAL ossification has been documented in cadaveric and imaging studies, its developmental mechanisms, biomechanical consequences, and clinical significance remain poorly understood. No studies to date have directly explored the impact of MAL ossification on lumbar spine function. Therefore, the objectives of this narrative, hypothesis-driven review are to (1) synthesise the existing anatomical, clinical, neurophysiological, and biomechanical literature relevant to MAL ossification; (2) propose three principal mechanistic pathways by which MAL ossification may contribute to chronic LBP; and (3) highlight implications for clinical assessment, imaging strategies, and therapeutic stratification in patients with chronic, non-specific LBP.

## 2. Methodology

### 2.1. Study Design

This study was conducted as a narrative, hypothesis-driven review aimed at synthesising the anatomical, biomechanical, neurophysiological, and clinical literature relevant to the lumbar MAL and its potential role in chronic LBP.

This review integrates foundational anatomical descriptions (e.g., Bogduk (1981) [16]; Maigne et al. (1991) [19]), contemporary morphological studies (e.g., Poodendaen et al. (2024) [5]), biomechanical investigations (e.g., Inoue et al. (2020) [6]; Panjabi (1992) [26]), and clinical research addressing facetogenic pain and medial branch interventions (e.g., Dreyfuss et al. (2000) [27]; Schneider et al. (2020) [28]; Cohen et al. (2020) [29]).

### 2.2. Search Strategy

A structured literature search was performed using the following electronic databases:

- PubMed/MEDLINE.
- Scopus.
- Web of Science.

Search descriptors included combinations of:

- “mamillo-accessory ligament”.
- “mamillo accessory ligament ossification”.
- “mamillo-accessory foramen”.
- “medial branch dorsal ramus”.
- “facet joint pain”.
- “facetogenic pain”.
- “lumbar spine biomechanics”.
- “paraspinal muscle denervation”.
- “multifidus atrophy”.
- “radiofrequency neurotomy”.
- “lumbar dorsal rami anatomy”.

Boolean operators (AND/OR) were applied to refine the search and combine anatomical, biomechanical, and clinical domains. Reference lists of included articles were manually screened to identify additional relevant studies.

### 2.3. Search Period and Language

The search included research published from database inception to January 2026, restricted to articles published in English.

### 2.4. Eligibility Criteria

Included studies comprised:

- Cadaveric anatomical studies examining the MAL, mamillo-accessory foramen, lumbar dorsal rami, and medial branch anatomy (e.g., Bogduk 1981 [16]; Maigne et al. 1991 [19]; Liu et al. 2024 [30]).
- Radiological investigations (CT- and MRI-based morphology studies) evaluating MAL ossification and facet degeneration [19,31].
- Clinical studies addressing medial branch pathology, facetogenic pain, and outcomes following medial branch blocks or radiofrequency neurotomy (e.g., Dreyfuss et al. 2000 [27]; Juch et al. 2017 [32]; Schneider et al. 2020 [28]; Cohen et al. 2020 [29]).
- Biomechanical analyses of lumbar segmental stability, facet joint loading, and finite element modelling (e.g., Panjabi 1992 [26]; Kuo et al. 2010 [33]; Park et al. 2025 [31]).
- Studies evaluating paraspinal muscle morphology, multifidus atrophy, and neuromuscular control in LBP (e.g., MacDonald et al. 2006 [34]; Fortin & Macedo 2013 [35]; van Dieën et al. 2019 [36]).
- Reviews on ligament ossification and spinal degenerative pathology, including the anterior [22] and posterior longitudinal ligaments [20], as well as ligamentum flavum ossification [21], where relevant to neural compression mechanisms.

Exclusion criteria included:

- Animal-only studies unrelated to lumbar biomechanics or neural compression mechanisms.
- Conference abstracts without full text availability.
- Non-peer-reviewed sources.
- Studies unrelated to lumbar spine anatomy, medial branch innervation, or facet-mediated pain.

### 2.5. Data Synthesis

Given the hypothesis-generating aim of this review, data were synthesised qualitatively using a structured narrative approach integrating the anatomical, biomechanical, neurophysiological, and clinical literature. Relevant studies were identified through targeted searches of anatomical, biomechanics, and spine-focused research to capture evidence pertaining to MAL morphology, medial branch anatomy, lumbar stability models, and facet-mediated pain. No quantitative synthesis or meta-analysis was performed.

Anatomical studies were evaluated to define MAL morphology, ossification patterns, and their spatial relationship to the medial branch of the dorsal ramus (MBDR), with attention to structural conditions that could permit neural entrapment. The biomechanical literature was examined within established spinal stability frameworks to assess how posterior element alterations may influence load-sharing, segmental control, and paraspinal muscle function. Clinical studies addressing facetogenic pain, multifidus degeneration, and medial branch-targeted interventions were appraised to determine translational plausibility.

The integrated evidence was used to develop an anatomically and functionally coherent mechanistic model linking MAL ossification with potential MBDR irritation, neuromuscular dysfunction, and altered lumbar kinetics. This synthesis is interpretive and intended to establish structural plausibility and guide future empirical investigation.

## 3. Emerging Mechanistic Domains for MAL-Associated Low Back Pain

This study examines how ossification of the MAL may disrupt lumbar spine biomechanics through mechanisms involving the muscular, neural, and articular subsystems. Sections are presented as thematic mechanistic domains grounded in established anatomical and biomechanical evidence, with clearly indicated interpretive extensions where conceptual inference is proposed. Specifically, MAL ossification may compromise the attachment and function of intrinsic spinal muscles, interact with adjacent neural structures, and restrict normal facet joint mobility.

In vitro studies have demonstrated the inherent instability of the lumbar spine, which relies on the coordinated interplay of the active (muscular), passive (osseous and ligamentous), and neural control subsystems to maintain stability and enable controlled motion [26]. Disruption to any of these subsystems has been shown to contribute to segmental instability, altered postural dynamics, and compensatory loading patterns throughout the lumbar spine. Given the multifactorial anatomical relationships of the MAL, three mechanistic domains are discussed below:

**Domain 1** : Muscular Attachment Interface and Segmental Stability

**Figure 1:**
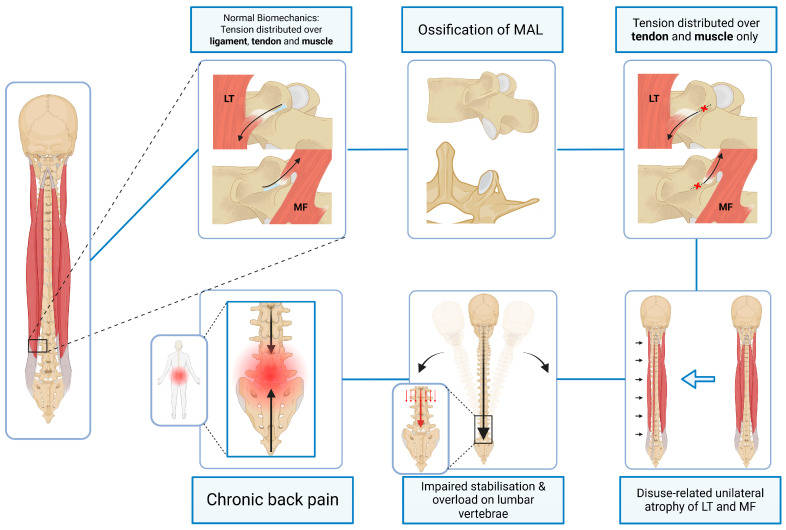
Schematic illustration of Domain 1 depicting the proposed muscular attachment mechanism by which ossification of the mamillo-accessory ligament (MAL) may influence segmental stability and contribute to genesis of chronic low back pain. LT = longissimus thoracis. MF = multifidus. (Figure was created by the authors using BioRender).

As previously stated, the MAL is a fibrous band of dense regular connective tissue that extends from the mammillary process to the accessory process of most lumbar vertebrae. The dorsal surface of the MAL provides an anchoring site for the lateral fascia of the lumbar multifidus (LM), a key stabiliser of individual lumbar spinal segments. The lateral surface of the MAL is also associated with the medial fibres of the longissimus thoracis (LT), which insert into the mammillary process, often traversing the MAL [16,17] (see Figure 1). These muscles contribute to dynamic segmental control and are essential for postural stability in the lumbar region [34,37].

Established anatomical and clinical evidence:

Ultrasound studies in individuals with acute unilateral low back pain demonstrate that the LM is, on average, 31% smaller on the symptomatic side compared to the contralateral side, with atrophy sharply localised to the single spinal segment corresponding with the patient’s clinical symptoms [38]. In patients with chronic low back pain (CLBP), bilateral LM atrophy, fatty infiltration, and structural degeneration are consistently reported [39,40]. Furthermore, individuals with CLBP exhibit impaired voluntarily activation of the affected multifidus region [41]. These findings collectively demonstrate that segmentally specific neuromuscular dysfunction is strongly associated with, and may contribute to, both acute and chronic lumbar pain states [42].

Panjabi’s spinal stability model posits that spinal stability is maintained through the coordinated action of three subsystems: the passive, active, and neural control systems [26]. Dysfunction in any one subsystem places greater demands on the other subsystems, potentially initiating a cycle of both overload and degeneration [37]. Finite element modelling of the L2–L3 segment has demonstrated that spinal asymmetry results in uneven load distribution and increased risk of degenerative change [33].

Interpretive extension:

Ossification of the MAL may alter the biomechanical interface between muscle and bone by reducing the compliance of the attachment site and replacing a flexible connective interface with a rigid osseous surface. Such alteration could modify tendon excursion, subtly influence the effective line of action of LM and LT fibres, or alter force transmission characteristics across the motion segment. Over time, these changes could theoretically contribute to altered recruitment patterns, asymmetric loading, and segmental instability (illustrated in Figure 1). Although direct biomechanical studies examining this specific muscle–ligament–bone interface are currently unavailable, this interpretation is mechanistically consistent with established relationships between multifidus dysfunction, asymmetric loading, and lumbar degeneration.

**Domain 2** : Neural Interface—Medial Branch of the Dorsal Ramus (MBDR)

At each segmental vertebral level, the spinal cord gives rise to a mixed spinal nerve composed of efferent fibres from the ventral root and afferent fibres from the dorsal root. After exiting via the intervertebral foramina, each nerve divides into ventral and dorsal rami [43]. The dorsal ramus courses through the intertransverse space between the medial and lateral intertransversarii lumborum, and gives rise to medial and lateral branches, and in some instances, an intermediate branch [16,43,44]. The lateral branch continues anteriorly, while the intermediate branch (when present) runs dorsally and caudally through the longissimus thoracis muscle [43].

The medial branch courses dorsally around the base of the superior articular process, passes through the groove formed by the junction of the roots of the transverse and superior articular processes. It then courses medially through a groove between the mamillary process and accessory process, deep into the MAL. The MBDR then runs medially and caudally over the lamina, deep into the multifidus muscle. Along its distal course, it gives branches to the zygapophyseal joints, as well as interspinous branches which innervate the interspinous ligament and interspinal muscles [30,45,46,47,48]. More importantly, the MBDR also gives off muscular branches that innervate key postural muscles of the lumbar spine, including the multifidus, intertransversarii mediales, and portions of the longissimus thoracis [16,47].

Due to the MAL’s trajectory between two features of the same vertebrae, it frequently forms an osseo-fibrous tunnel guiding the MBDR [16,49]. When ossification occurs, a partial or complete bony bridge (mamillo-accessory foramen) may form, encircling the nerve [5,17,18].

Established clinical and anatomical evidence:

Compression of spinal nerves by ossified ligaments is not without precedent. Analogous conditions, such as thoracic outlet syndrome or entrapment of the superior cluneal nerve, have been implicated in unexplained chronic pain syndromes that only resolve once the anatomical cause is specifically identified and addressed [50,51]. This anatomical configuration is rarely considered in clinical evaluations, despite the potential for significant neurovascular compromise.

Chronic nerve compression has been shown to progress from numbness or paraesthesia to neuropraxia, muscle weakness and atrophy with associated axonal degeneration [52,53]. Studies investigating cohorts with non-specific LBP consistently demonstrate paraspinal muscle degeneration, including reduced cross-sectional area and altered composition [35,39,40,54]. These findings underscore the importance of intact neural input for maintaining paraspinal muscle integrity and the association with the development and persistence of LBP [39,55].

Analogous entrapment syndromes, such as superior cluneal nerve compression or thoracic outlet syndrome, illustrate how subtle anatomical constraints may generate persistent pain until specifically identified and addressed [50,51,56].

**Figure 2:**
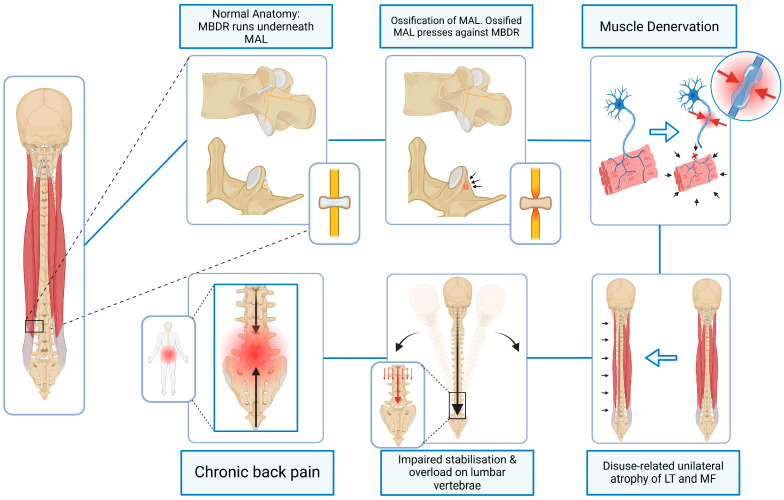
Schematic representation of Domain 2 illustrating the anatomical relationship between an ossified MAL and the MBDR and their potential contribution to paraspinal muscle atrophy. LT = longissimus thoracis. MF = multifidus. (Figure was created by the authors using BioRender).

Interpretive extension:

In cases of partial or complete MAL ossification (type II or III), reduction in the available osseo-fibrous space may increase the likelihood of mechanical irritation or low-grade compression of the MBDR. If unilateral, such irritation could plausibly contribute to asymmetric neuromuscular activation and progressive muscle imbalance (see Figure 2). While direct clinical evidence confirming MBDR entrapment beneath an ossified MAL remains limited, the anatomical configuration and established pathophysiology of chronic nerve compression render this mechanism anatomically and biologically plausible, though not yet empirically confirmed.

**Domain 3** : Pain Modulation and Secondary Postural Adaptation

**Figure 3:**
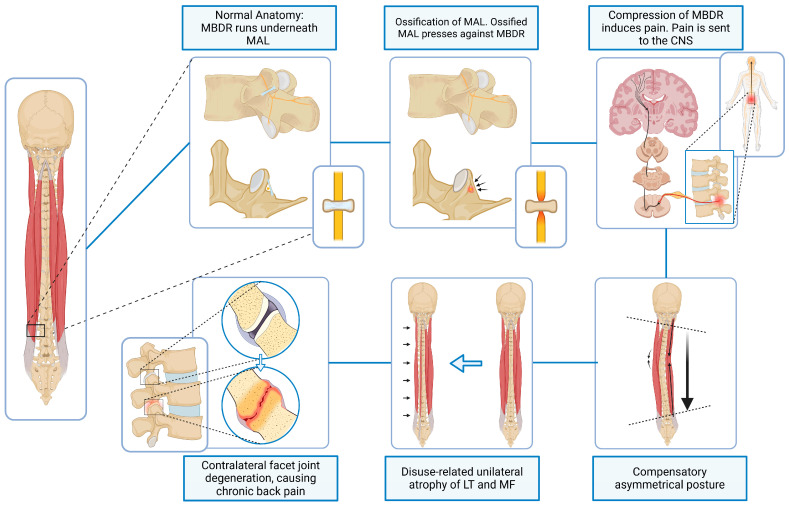
Schematic of Domain 3 depicting how ossification of the mamillo-accessory ligament (MAL) may compress the medial branch of the dorsal ramus (MBDR), resulting in pain and compensatory postural changes in the paraspinal muscles that could contribute to altered lumbar biomechanics. LT = longissimus thoracis. MF = multifidus (Figure was created by the authors using BioRender).

The MBDR carries afferent fibres from the zygapophysial joint capsule and adjacent soft tissues, making it a key structure in both the pathophysiology and treatment of facetogenic LBP [10,57]. Persistent irritation of the MBDR may increase sensitivity of facet joint sensory nerves and amplify nociceptive signalling [5,17,58] (Figure 3).

Established clinical evidence:

Altered postural control, asymmetric muscle activation, reduced range of motion, and aberrant loading patterns are consistently observed in chronic LBP populations [36,59,60,61,62,63,64,65]. These adaptations frequently arise through a feedback loop of pain and sensorimotor response aimed at protecting the affected region [62,63,64,65]. Although initially protective, such adaptations have been shown to introduce compensatory loading patterns that may perpetuate biomechanical dysfunction [31,33,35,39,64,65,66].

**Figure 4:**
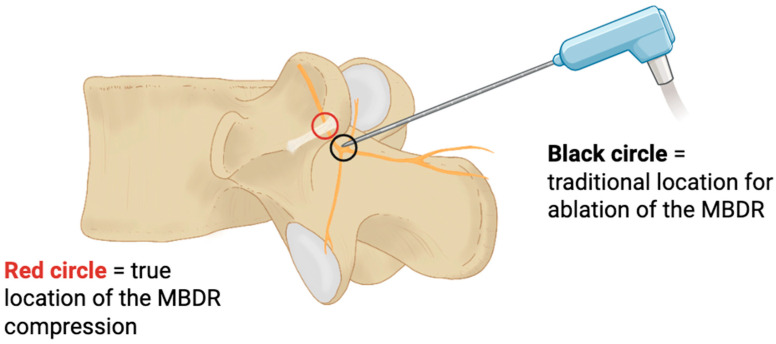
Schematic representation of the conventional target site for ablation of the medial branch of the dorsal ramus (MBDR) (black circle). While this approach denervates the articular branches supplying the facet joint capsule, it does not address the more proximal segment of the MBDR, where compression beneath the mamillo-accessory ligament (MAL) is likely to occur and may represent the true nociceptive source (red circle).

Clinically, this hypothesis may help explain cases in which patients present with chronic unilateral axial back pain, segmental stiffness, or resistance to conventional treatments such as radiofrequency ablation (RFA). Although RFA can effectively reduce facet-mediated pain in many patients, it is subject to well-recognised limitations. The technique relies heavily on accurate needle placement and lesioning strategy, and pain often recurs within months; some patients do not experience meaningful relief at all [28,32] (see Figure 4).

RFA is designed to interrupt the nociceptive input from the MBDR. However, its failure in certain patients, despite anatomically precise targeting, may reflect ossification of the MAL and consequent entrapment of the MBDR as an overlooked anatomical contributor. In this scenario, limited RFA efficacy may be attributable to the location of the lesion relative to the site of entrapment. If symptoms arise from compression of the MBDR beneath the MAL, then ablating the nerve distal to this region would leave the proximally compressed segment unaffected. As a result, nociceptive signalling may persist despite technically successful lesioning, accounting for ongoing or recurrent pain.

As ossification of the MAL is not routinely assessed on standard imaging and is often overlooked even in cadaveric dissection, its role as a potential pain generator has likely been underreported and, thus, not considered in a surgical or clinical context. This may explain why some patients do not respond to conventional treatments like medial branch blocks or RFA, even when these procedures are technically well executed [27,29]. Despite this possibility, the role of MAL ossification in neural compression remains poorly explored in the current literature.

## 4. Implications of the Proposed Hypotheses

Ossification of the MAL is a relatively common anatomical finding, particularly at L3–L5, and its prevalence increases with age. Concurrently, LBP remains the leading global cause of disability, yet many cases are poorly explained or unresponsive to conventional treatments. RFA, a standard intervention for facet-mediated pain, fails in a notable subset of patients despite accurate targeting, suggesting that previously unrecognised anatomical or biomechanical factors, such as MAL ossification, may contribute to treatment resistance.

Muscular asymmetries, particularly in the LM, are frequently observed in individuals with chronic LBP, often without clear explanation. Evidence from imaging and morphometric studies demonstrates focal LM atrophy, fatty infiltration, and impaired voluntary activation, supporting a role for compromised spinal stabilisers in LBP pathophysiology [39,40,41,54]. Integrating these observations with the mechanistic domains described above, ossification of the MAL may mechanically and/or neurologically disrupt key spinal stabilisers and sensory pathways, contributing to segmental dysfunction. Clinically, this framework may help explain otherwise enigmatic presentations of unilateral pain, segmental stiffness, or failed interventions.

### 4.1. Clinical Assessment and Identification of the MAL

MAL ossification is rarely visualised on standard imaging and is often overlooked even in cadaveric studies. When clinically suspected, identification strategies include high-resolution CT or MRI to visualise bony bridges or foramen formation, ultrasound to evaluate muscle–ligament interfaces, and detailed physical examination for segmental stiffness or asymmetrical posture. Additional diagnostic approaches may include medial branch blocks or nerve conduction studies to assess potential entrapment of the MBDR. These methods provide a framework for clinicians to integrate anatomical findings with functional impairment, as highlighted in established musculoskeletal modelling and decision-making frameworks [5,16,27] (summarised in Table 1).

### 4.2. Treatment Approaches

A spectrum of interventions has been proposed or utilised to address MAL-associated or related dysfunctions:

- Physiotherapy and targeted exercise: Focused strengthening of the LM, multifidus, and paraspinal stabilisers, as well as postural retraining, may partially compensate for structural constraints and improve segmental control [34,35,36,42,63,64].
- Pharmacological management: NSAIDs, muscle relaxants, and analgesics may provide symptomatic relief, though they do not address the underlying mechanical or neurological disruption [11,13,58,67,68].
- Interventional pain procedures: RFA or medial branch blocks can reduce facet-mediated pain but may fail if MAL-related entrapment is unrecognised. Adjustments in target site selection informed by imaging or modelling may improve outcomes [4,24,27,28,29,32,57].
- Surgical considerations: Surgical decompression is well described for other ossified spinal ligaments [50,51]; however, specific reports addressing ossified MAL are limited and largely anatomical or anecdotal in nature [5,17].

With specific respect to pharmacological management, it must be emphasised that these pharmacological strategies are primarily symptom-modifying rather than disease-modifying interventions. In the context of the proposed mechanistic domains of MAL ossification, such agents do not reverse structural ossification, alleviate potential medial branch nerve entrapment, or restore altered biomechanical load distribution. Their role is therefore supportive, aimed at facilitating participation in active rehabilitation strategies rather than addressing a putative anatomical driver of pain.

Furthermore, current pharmacological recommendations for chronic LBP are based on heterogeneous patient populations and are not specific to MAL-related pathology. There are no randomised controlled trials evaluating pharmacological efficacy in patients with confirmed MAL ossification. Consequently, any pharmacological approach in this context should be interpreted within established chronic LBP guidelines and individualised according to risk–benefit considerations, comorbidities, and duration of use. Long-term reliance, particularly on opioids, is not supported by high-quality evidence and carries well-recognised safety concerns.

## 5. Testable Predictions and Future Research Directions

To transition the conceptual framework of this narrative review towards empirical evaluation, the proposed mechanistic domains generate several testable, falsifiable research predictions that can be systematically investigated using contemporary imaging, physiological, and modelling techniques.

### 5.1. Imaging–Symptom Correlation

Prospective clinical studies using high-resolution CT and MRI should investigate whether the presence, morphological type (e.g., MAN vs. MAF), or size of MAL ossification correlates with specific clinical features such as unilateral axial pain, multifidus asymmetry, or resistance to medial branch interventions. Previous cadaveric and radiological studies demonstrate that MAL ossification is common and increases with age [5], and that the medial branch courses beneath the MAL in a consistent anatomical relationship [16,30]. These studies should also aim to standardise imaging protocols and scoring systems to improve reproducibility and enable correlation with clinical and functional outcomes.

### 5.2. Quantitative Morphometric Analysis

Standardised morphometric assessments of the MAF and surrounding osseo-fibrous corridor should be performed to establish normative ranges and thresholds associated with neural proximity or potential mechanical interaction with the medial branch of the dorsal ramus (MBDR) [69]. By adapting objective CT-based metrics, this approach can complement imaging–symptom studies, reduce redundancy, and identify anatomical features most predictive of neuromuscular compromise.

### 5.3. Neurophysiological Assessment

Electrodiagnostic techniques, including electromyography (EMG) and nerve conduction studies [70], could evaluate whether patients with advanced MAL ossification show segmentally specific changes in medial branch function or multifidus activation. These assessments should be integrated with morphometric and imaging data to determine whether structural alterations translate into functional deficits, reducing repetitive emphasis on morphology alone.

### 5.4. Biomechanical Modelling

Finite element modelling and other computational approaches should simulate how variable MAL morphologies influence load sharing, segmental stiffness, and intervertebral kinetics [17]. Incorporating findings from imaging, morphometric, and neurophysiological studies into the models can reduce duplication of effort and generate testable predictions about how ossification might contribute to mechanical instability or asymmetric load distribution.

### 5.5. Interventional Outcome Stratification

Subgroup analyses of outcomes following medial branch radiofrequency ablation (RFA) or diagnostic blocks could determine whether MAL morphology predicts therapeutic response [28]. This strategy synthesises the above data, linking structural, functional, and clinical dimensions to guide personalised interventions and avoid repeating separate recommendations for each domain.

## 6. Conclusions

**Figure 5:**
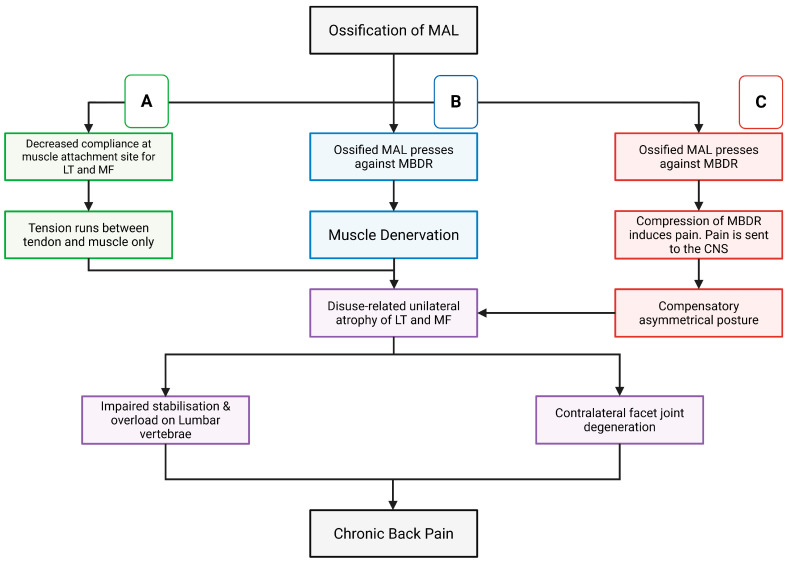
Flowchart summarising how the three proposed mechanistic domains may contribute to paraspinal muscle atrophy, facet joint degeneration, segmental destabilisation, mechanical overload, and chronic low back pain. Boxes are colour-coded to indicate relevance to Domain 1 (green), Domain 2 (blue) or Domain 3 (red). Purple boxes represent the sequence of potential downstream outcomes triggered in response to these domains. LT = longissimus thoracis. MF = multifidus (Figure was created by the authors using BioRender).

This narrative review synthesises anatomical, biomechanical, and clinical evidence to propose three mechanistic domains, muscular, neural, and adaptive, through which MAL ossification may plausibly contribute to chronic low back pain (please refer to schematic shown in Figure 5). Although MAL ossification is a documented anatomical finding, its functional and clinical significance remains uncertain.

By consolidating prior recommendations, future research should focus on integrated studies combining imaging, morphometric analysis, neurophysiological assessment, biomechanical modelling, and interventional outcome stratification. Until such data are available, MAL ossification should be considered a potential anatomical modifier rather than a confirmed pain generator.

## 7. Limitations and Final Considerations

Direct experimental or prospective clinical validation of MAL ossification as a causal factor in LBP remains lacking. Limitations include heterogeneity of imaging and morphometric studies, absence of longitudinal data, and potential confounders such as age-related degeneration, generalised osteoarthritis, or other spinal variants. Future research should prioritise integrated, multimodal approaches to reduce redundancy, directly link structure to function, and clarify clinical relevance.

In conclusion, ossification of the MAL represents a potentially underappreciated anatomical contributor to chronic LBP, capable of producing muscular, neural, and biomechanical alterations. Recognising and assessing this structure in clinical practice may refine diagnostic accuracy and improve treatment outcomes for individuals with persistent LBP.

## Figures and Tables

**Table 1:** Conceptual framework linking mechanistic domains with clinical considerations.

Mechanistic Domain	Plausible Clinical Features *	Diagnostic Considerations ^†^	Management Approaches (Extrapolated from LBP Guidelines)
**Domain 1**: Muscular Attachment Interface	Segmental stiffness; focal multifidus asymmetry; altered recruitment patterns	MRI morphometric assessment; ultrasound evaluation; CT assessment of MAL morphology	Motor control retraining; segmental stabilisation; progressive strengthening programmes
**Domain 2**: Neural Interface (MBDR)	Unilateral axial pain; partial response to medial branch block; paraspinal asymmetry	High-resolution CT; targeted MRI; diagnostic medial branch block	Medial branch block; RFA (standard technique); multimodal rehabilitation
**Domain 3**: Pain Modulation and Postural Adaptation	Persistent asymmetric loading; reduced range of motion; recurrent symptoms	Functional movement assessment; dynamic load analysis	Multimodal management including exercise therapy, short-term NSAIDs, and selected interventional procedures

* Clinical features are non-specific and commonly observed in chronic LBP. **^†^** No imaging or clinical modality currently confirms MAL-related pain causality.

## Data Availability

All data are reported in the published version of this article.
